# What works? A grounded theory investigation of training non‐psychology staff in using Solution‐Focused Brief Therapy

**DOI:** 10.1111/papt.70009

**Published:** 2025-08-17

**Authors:** Haakon Juul, Dominic Bray, Ian C. Smith

**Affiliations:** ^1^ Division of Health Research Lancaster University Lancaster UK; ^2^ Mersey Care NHS Foundation Trust Prescot UK; ^3^ Present address: Department of Primary Care and Mental Health University of Liverpool Liverpool UK

**Keywords:** brief therapy training, grounded theory, medical settings, multidisciplinary staff, non‐psychology staff, Solution‐Focused Brief Therapy

## Abstract

**Objective:**

As part of a task‐sharing strategy, clinical psychologists are becoming increasingly expected to offer therapy training for staff in health care services to develop psychological mindedness to increase access and provision of psychological support for clients. The current study explored how 10 staff working in health care settings experienced Solution‐Focused Brief Therapy (SFBT) training and how they subsequently used it.

**Methods:**

One‐to‐one semi‐structured interviews were conducted with 10 participants; a constructivist grounded theory (GT) approach was used to generate a model based on the participants' reflections after the training.

**Results:**

Staff shared how they felt they needed evidence of SFBT effectiveness in order to believe that learning a new model would be worth the required investment. They also found realistic role modelling that was relevant to their context to be particularly convincing as well as regular support from their peers and multidisciplinary meetings. Participants also shared some barriers to using SFBT in practice, including time‐restricted clinics, service pressures and challenging clients.

**Conclusion:**

The model describes a complex dynamic between personal, interpersonal and systemic factors that influenced the staff members' individual decision to abandon the more familiar medical model that represented a sense of comfort and safety. The study includes recommendations for how clinical psychologists can address the identified facilitators and barriers to facilitate more effective training programmes and training transfer.

## INTRODUCTION

In the UK's primary care services, it is estimated that 20–25% of consultations and medical clinics are provided for people with chronic or otherwise debilitating health symptoms with no known medical cure (Hartman et al., 2013; Knapp, McDaid and Parsonage, 2011). Individuals presenting with such symptoms, such as diabetes and chronic pain, often benefit significantly from psychosocial support which motivates them to use behavioural management strategies, improving their psychological well‐being (Mental Health Taskforce, 2016). Despite this, the majority of these patients in the UK are only offered medical treatment (Mind, 2011), due to psychosocial difficulties not being identified in medical appointments, leading to misdiagnosis and deterioration of mental health with patients subsequently requiring input from secondary care services where there are long waiting lists (Negri et al., 2021). There is therefore a need for improved integration between mental and physical health services to address these issues.

One approach to facilitate this has been for clinical psychologists to prioritise consultation, supervision and training to increase the availability of psychological support and enable other healthcare professionals to deliver more effective and coordinated psychological interventions in primary and community care settings.

This strategy, known as task‐sharing (Hoeft et al., 2018), aims to increase the availability of psychological support by redistributing resources from specialist psychological practitioners to non‐specialist, non‐psychological health care professionals. One way in which clinical psychologists can facilitate task‐sharing is by providing therapy training to health and social care staff who do not work in the psychological professions. However, in order for this to be effective in medical settings, it is important to identify a model of therapy suitable to the medical context (Blount, 2003). Researchers have argued that psychological interventions that can demonstrate effectiveness whilst also incorporating brevity to meet the patient's physical abilities and time constraints would be ideal (Cunningham, 2009; Olfson et al., 2013; Zhang et al., 2018).

There is now a large body of data demonstrating that shorter‐form therapies are comparable with longer‐term therapies in terms of outcomes, including symptom improvements and treatment gains over time (e.g. Abbass et al., 2014; Howard et al., 1986; Johnson & Gelso, 1981; Lambert & Bergin, 2013, pp. 3–5). This has led to a shift towards developing condensed, evidence‐based treatments with an average of only 6–10 sessions, and to the broad adoption of this kind of intervention within the UK (Cape et al., 2010).

A recent accumulation of evidence has demonstrated that non‐psychology staff can deliver therapy successfully using a range of brief therapies, even after only a small amount of training. Some of this evidence comes from randomised controlled trials (RCTs) that have reported outcomes for healthcare staff delivery in some situations can be comparable to those for specialist staff, even after being trained for only five (Ekers et al., 2013) or as few as two (Jahoda et al., 2017) days. These outcomes included symptom reduction and improvements in Routine Outcome Monitoring (ROM) measures. The models used in such studies have included Brief Cognitive Behavioural Therapy (BCBT) (Cully et al., 2017), Behavioural Activation (Ekers et al., 2013) and Motivational Interviewing (Bennett et al., 2007; Carroll et al., 2006).

Another such brief model, which has also been implemented by a variety of health care staff not from the psychological professions (e.g. Hosany et al., 2007; Simm, 2013; Kim et al., 2017), is Solution Focused Brief Therapy (SFBT). SFBT is a short‐term, goal‐oriented approach that aims to build solutions by tapping into the clients' own resources and strengths in order to support them to achieve and sustain desired behavioural change (de Shazer et al., 1986; Trepper et al., 2006). By identifying what already works for the client, the approach does not require an in‐depth exploration of the past or the use of psychological formulations in order to resolve the presenting problem. Instead, the focus is on identifying current strengths, clarifying the preferred future and developing client motivation so they are able to move past current difficulties using their own strategies. This makes it distinct from other briefer and manualised approaches where formulation is a central part of the therapy. The clinician can therefore support the client to be the expert (e.g. Bray, 2022), using questioning techniques to identify exceptions to their difficulties and define ‘preferred futures’ that lead to lasting and adaptive solutions (Iveson, 2002). The model has amassed a considerable evidence‐base demonstrating recurrent positive outcomes for a range of clients, with various presentations including people with longstanding physical health conditions (Franklin, 2015; Gingerich & Peterson, 2013; Kim et al., 2019).

In addition to the evidence‐base around therapy outcomes, SFBT has also been proposed as a model particularly suitable for health care practitioners to utilise, as it requires little previous knowledge of psychology or therapy and requires less time to implement in practice, making it more cost‐effective (Hosany et al., 2007). This has been supported by qualitative studies reporting the model as being easy to understand and implement (Bowles et al., 2001; Smith & Macduff, 2017) as well as adaptable to contexts such as on‐call shifts on inpatient wards (Blayney et al., 2014) and in medical services for stroke and pain (Simm, 2013; Simm & Barker, 2018), diabetes management and other health management services (Gingerich & Peterson, 2013; Kim, 2007). Furthermore, the model has been espoused as particularly useful for services offering support for individuals with long‐term conditions that cannot be ‘fixed’ (Bray, 2009); it facilitates motivation and independence necessary for ongoing self‐management.

Another reason for its applicability to medical settings is that SFBT aligns well with the increasing recognition of client expertise, choice and empowerment in such services. This was emphasised in the ‘expert patient strategy’ outlined by the Department of Health (2001) in the UK, which proposes that ‘knowledge and experience held by patients has been for too long an untapped resource’. Finally, qualitative research has also found that the SFBT model generally fit well with the personal and professional values health care practitioners, particularly nurses, (Bowles et al., 2001; Cunanan & McCollum, 2006; Smith, 2010; Smith & Macduff, 2017; Stark et al., 2018) developing a form of ‘allegiance’ (Wampold, 2001) with the model.

However, despite some promising results from research, a variety of barriers have also been identified that could prevent health care practitioners from implementing SFBT in their practice. Many of these barriers are similar to those found in the general literature around successful implementation of skills following training. Kirkpatrick and Kirkpatrick (2006) identified four levels at which the training could be evaluated, including: (a) trainee satisfaction; (b) knowledge gain; (c) transfer of skills to practice; and (d) the effect of the training on outcomes. Of these four factors, transference of skills into practice is often seen as the most problematic to achieve, with studies demonstrating that staff tend to apply as little as 10–30% of the training content in practise (Nielsen & Shepherd, 2022). The same problem is seen within the therapy training‐specific literature, which indicates that many staff fail to apply techniques and strategies, despite demonstrating high rates of satisfaction and learning when being trained in them (e.g. Jahr, 1998; Milne et al., 2000). Several barriers to training transfer have been identified, including a lack of supportive organisational policies and managers, a lack of time in clinics, a lack of supervision and the trainees' perceptions of the applicability of a model to their client group and their ability to use it (e.g. Nielsen & Shepherd, 2022; Seko et al., 2021).

In the case of SFBT training, the above factors have also been found to be important in the transfer of skills (Hosany et al., 2007; Smith, 2010; Smith, 2011). In addition, however, other barriers such as managerial pressure to apply problem‐focused models (Smith, 2011), lack of confidence in using strength‐based approaches (Simm et al., 2011) and negative attitudes towards the model being formed after seeing it being used rigidly (Cunanan & McCollum, 2006) have also been identified as important. Furthermore, some findings indicate that implementation may not occur even in the presence of reported high affiliation with SFBT or motivation to apply techniques in practice (Cunanan & McCollum, 2006). Evidence from qualitative investigations suggests that more external barriers likely play a key role in preventing SFBT implementation.

Although some facilitators and barriers have been identified and replicated in the training literature so far, studies to date have not examined in detail how these factors interact in determining the form of implementation of SFBT in practice. By utilising a constructivist grounded theory approach to examine participants' experience and understanding in depth, in our study we were able to explore how the perceived barriers and facilitators emerging from our data were seen to interact and unfold following training of non‐psychology staff working in health care settings, and how this interaction might influence trainees' implementation of SFBT. We explored this in the context of brief training programmes (defined as 40 h or less (Smith, 2011; Stark et al., 2018)) provided by clinical psychologists. This approach was designed to produce findings to facilitate the development of more tailored training methods that can be adapted to different contexts, to maximise the long‐term efficacy of training (Volet, 2013).

## MATERIALS AND METHODS

### Participants

Ten health care staff working in five services across four national Health Service (NHS) Trusts in the UK participated in the study. Demographic and training details are included in Table 1. Names of the employing NHS Trusts have been withheld to protect participant anonymity.

**TABLE 1:** Demographic and training‐related information on participants in the study.

No.	Gender	Age	Title/role	Service	Year and month of most recent training	Interview year and month	Duration of most recent training course	No. of completed training courses	Virtual or F2F training	Optional v. required attendance	Formal clinical supervision
1	Female	57	Specialist Nurse	Frailty Service, Trust A	Jan 2021	Jan 2023	Two days	1	Virtual	Opted in	N
2	Female	62	Specialist Nurse	Chronic Pain Service, Trust B	Sep 2022	Jan 2023	Two days	1	F2F	Opted in	N
3	Male	29	Specialist Nurse	Paediatric Service, Trust C	Sep 2022	Jan 2023	Two days	1	F2F	Opted in	N
4	Female	46	Specialist Nurse	Diabetes Service Trust C	Sep 2022	Feb 2023	Two days	1	F2F	Opted in	N Some informal supervision for Clinical Psychologist
5	Female	49	Consultant Endocrinologist	Paediatric Service, Trust C	2019	Feb 2023	Two days	3–4	F2F	Required	Y SFBT‐specific for several year, ongoing.
6	Female	33	Specialist Mental Health Research Nurse	Psychological Intervention Service, Trust B	Sep 2022	Feb 2023	Two days	1	F2F	Required (mandatory)	Y Non‐SFBT supervision for several sessions over 6 months
7	Female	54	Specialist Research Nurse	Paediatric Service, Trust C	201	March 2023	Two days	1	F2F	Opted in	Y 4× SFBT sessions, 2 years after training. Informal supervision provided over a 6‐month period
8	Female	35	Specialist Nurse	Paediatric Service, Trust C	2021	March 2023	Two days	2	Virtual	Required (induction)	Y 4× SFBT sessions, 2 years after training. Informal supervision provided over a 6‐month period
9	Female	37	Specialist Nurse	Paediatric Service, Trust C	2021	March 2023	Two days	1	Virtual	Required (induction)	N
10	Female	48	Rehabilitation assistant	Chronic Pain Service, Trust C	2021	March 2023	Two days	1	Virtual	Required (induction)	N

Eligibility criteria were developed prior to recruitment and are detailed in Table 2.

**TABLE 2:** Inclusion and exclusion criteria for potential participants.

Inclusion criteria	Exclusion criteria
No formal experience of applying psychological therapy prior to receiving SFBT training	Accredited qualifications or apprenticeships in clinical psychology, counselling or coaching prior to receiving SFBT training
Training delivered or jointly delivered by a Clinical Psychologist, trainee clinical psychologist, or assistant psychologist under qualified supervision	Received SFBT training from non‐qualified professionals only, without supervision from a qualified psychological professional
Attended a primary SFBT training programmes of 40 h or less	Those who attended a primary SFBT training programme exceeding 40 h
At least 3 months of clinical experience following primary SFBT training	Less than 3 months of clinical experience following primary SFBT training

Potential participants were e‐mailed with information about the project by a colleague of the second author, who worked for the NHS Trust that had provided the training. Those who wished to participate were asked to contact the first author. Sixty‐five staff members were invited to participate, of whom 10 responded. All participants gave informed consent to participate in the study.

The recruitment was conducted in three phases: three participants were interviewed in an initial round of data collection, followed by a further three participants, and then a final four, with data analysis being conducted in between each phase, which then informed and shaped the subsequent interviews. All interviews were conducted by the first author via a virtual platform and were recorded.

Theoretical sampling was used to increase the richness of data as is recommended in GT. Two participants were chosen specifically in an attempt to reduce the homogeneity of the dominant female nurse participant group, including a male participant and a support worker (participant 3 and 10 respectively). The aim of the study was to use theoretical sampling to identify the most relevant participants that were suited to inform the research questions. However, due to limitations around recruitment, the opportunities for such sampling were limited; to compensate, a heavy focus was placed on theoretical questioning, whereby the interview schedule was adjusted as the interviews and analysis progressed in order to further explore both important topics that arose in more detail and gaps that were left following earlier interviews. Theoretical sufficiency was deemed to be achieved after 10 interviews, and so no further attempts at recruitment were undertaken (Corbin & Strauss, 2015; Dey, 1999, p. 257).

### Data collection

Data were collected from semi‐structured interviews that lasted from 50 to 110 min. The interview recordings were automatically transferred to a secure online server where they were transcribed and anonymised.

### Design and analysis

The study utilised a qualitative design, involving semi‐structured interviews. Data were transcribed verbatim and analysed using a constructivist grounded theory approach (Charmaz, 2014; Glaser & Strauss, 1973). This method was chosen due to its suitability for generating a new theory based on the experience that can encompass factors and processes that are poorly understood in the literature (Charmaz, 2014). Data were analysed iteratively using a constant comparative method to continuously compare emergent data in order to develop a theory relating to the research question (Charmaz, 2014). Analysis comprised line‐by‐line coding of the transcripts followed by focused coding, which were based on more salient information relating to the research question (Charmaz, 2014).

Once initial and focused coding was completed, conceptual codes were developed, which formed the basis of developing preliminary theoretical categories. Once all theoretical categories were formed, a core category was identified (‘the two states of mind’) which was pervasive across conceptual codes. This category was then used as a framework for the structure of a process model of the participants' experience of SFBT training which was developed.

The analysis process was aided by ongoing memo‐writing and free‐writing throughout (Charmaz, 2014). Furthermore, quotes were also used throughout the analysis and drafting of the results to keep the model grounded in the data. The constant comparison method was applied by looking for similarities and differences between the codes and categories of all interviews throughout the analysis process (Charmaz, 2014).

Finally, the study adopted a constructivist approach, assuming that theories are constructed through the research process (Charmaz, 2014). Therefore, the grounded theory from the study is regarded as an interpretative representation, not an objective ‘truth’. This way, the researchers' own biases and interpretations will have impacted the outcomes of this research project, as this is unavoidable despite any efforts to reduce any bias (Charmaz, 2014).

### Ethical permission

Ethical permission was granted by Lancaster University and the UK NHS Health Research Authority (HRA). Due to its personal and qualitative nature, the data collected in this study cannot be made publicly available.

### Reflexivity and credibility

The first author, who collected the data and conducted the primary analysis, was a trainee clinical psychologist with no previous clinical or research experience around SFBT, but who had attended two introductory teaching sessions on the therapy as part of their professional training. To reduce the impact of bias, the first author minimised reading of any relevant literature around the project until the data was collected, in line with GT guidance. They also utilised a reflective diary, documenting any assumptions or potential pre‐conceived ideas relating to the research throughout the project.

The second and third authors had secondary and supervisory input into the later stages of the analysis. Both are experienced clinical psychologists with extensive experience of clinical practice using SFBT and providing training in the model to others. The threat of bias was also discussed in supervision, where the interview and analysis process were checked to minimise preconceptions and leading questions.

## RESULTS

**FIGURE 1:**
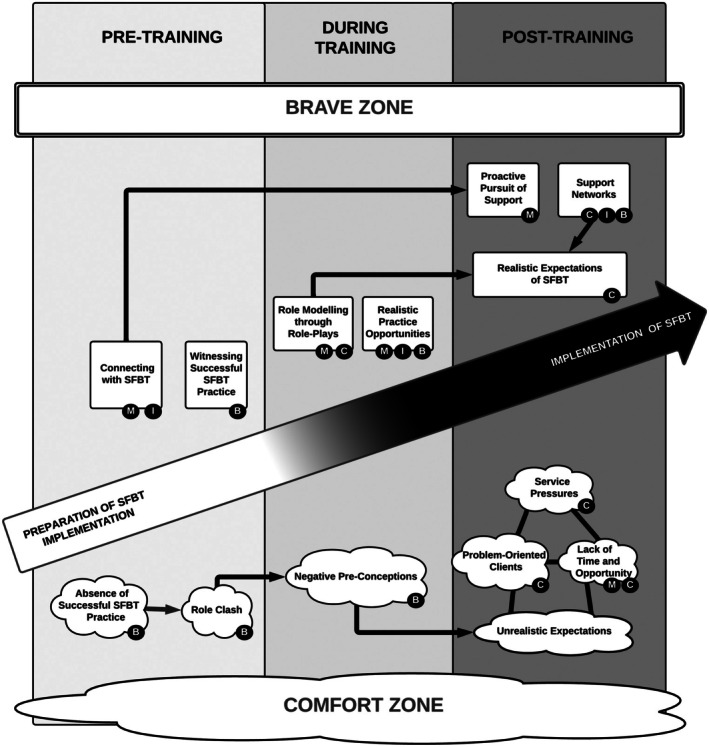
Model of SFBT implementation in clinical practice. The internal states of processing that are influenced by each theme are indicated using labelled black spots, where B = belief, C = Confidence, I = Interest, and M = Motivation. All of these factors were seen as interacting with each other when they co‐occurred within a stage of implementing Solution Focused Brief Therapy (SFBT).

As part of the analysis, a model of SFBT implementation in clinical practice was developed (see Figure 1). In the figure, a successful process of implementation is depicted by the diagonal arrow. The analysis indicated that this optimal trajectory is seen as depending on the interaction between four separate elements consisting of: (1) a core category consisting of two opposing mental states, (2) a sub‐category of four internal experiential states, (3) 13 themes of facilitators and barriers, and (4) three chronological stages of implementing SFBT. These elements together describe the process of how participants are pulled into or pushed away from implementing SFBT and will be introduced in their respective order.

### Core category: The two states of mind

This category describes two states of mind that participants were in throughout the process of using SFBT in practice. The first category, ‘Comfort Zone’, can be seen at the bottom of the figure, and relates to a state of mind that has been described by participants as ‘comfortable’ and ‘familiar’ and being ‘where your experience lies’ (Participant 4, 7). All participants described the comfort zone as being associated with using a ‘medical model’ that they were familiar from their training and gave them a sense control when using it. Participants stated that since the goals of the medical approach would often contrast that of SFBT, they felt using one would often exclude the other. The second category, found at the top, just below the four stages, is the ‘Brave Zone’. Being in this state of mind was often described with a feeling of ‘discomfort’, but with a willingness to learn something new, therefore often referred to as a ‘sacrifice’ worth the effort: ‘so it's not comfortable for me at that moment in time, but… I still feel that it's worth doing because it can make a difference where other things haven't made a difference’ (Participant 7).

Participants described various aspects of their working environment acting as factors that either pulled them into using the medical rather than the SFBT model or vice versa. This ‘push and pull’ dynamic is represented by the upwards and downwards arrows (in Figure 1) and are affected by the four internal states.

### Sub‐category: The four internal states of processing

This sub‐category describes four internal states of processing (illustrated as the smaller black circles which are used to label the themes) that are involved in determining the outcome of the SFBT learning process. These are (1) Interest, indicated with the letter I, which relates to an interest in the SFBT model and training, (2) Motivation (M), which relates to the intent to act upon stated interest, whether it would be motivation to attend or engage in the training or motivation to use SFBT in clinical practice, (3) Belief in SFBT (B) which relates to how much the participants believed that SFBT is an effective, useful and applicable model, and (4) Confidence (C) which relates to the participants' perceived ability to implement SFBT in practice. Within each of the stages of implementing SFBT, the states of internal processing that were present were seen by participants as interacting with each other.

Although a careful analysis of the data indicated that all four factors were directly involved in explaining the relationship between various influences and SFBT implementation, it did not suggest that they were all needed to ensure it. For instance, several participants described times when they experienced minimal confidence but still made attempts to use SFBT, for example: ‘I think when we first started our confidence and knowledge of it was a bit ropey…but we were motivated to give it a go’ (Participant 3).

Therefore, these factors should mainly be understood as a combination of multiple partial mediating variables that together alter the degree of SFBT implementation. This way, staff require at least one internal state (e.g. motivation) to be experienced to use SFBT, but will also be significantly more likely to use it if all are experienced. Finally, the influential strength of these variables was seen as being dependent on the facilitators and barriers as introduced below.

### The 13 themes seen across the three stages of implementing SFBT

This part of the model depicts the process of SFBT learning in a chronological fashion, starting from when participants heard about SFBT to ‘today’, when participants had been using the model for up to several years. The stages are pre‐training, during, and post‐training (see top of Figure 1). Each stage included themes (located above or below the circle to signify facilitators (above) and barriers (below)) that influenced the states of processing, which then influenced the next stage. For instance, an overall positive experience of SFBT in the pre‐training stage (e.g. high interest and motivation and positive belief) would likely facilitate training attendance, engagement, and an overall positive training experience, which again would likely facilitate the process of implementing SFBT in practice. Some of the themes were seen to influence each other, which is either illustrated with black arrows (indicating perceived causal links) or lines (indicating correlation). Finally, the themes are marked with labelled spots signifying which of the four internal states they influence.

#### Stage 1: Pre‐training

The first stage, along with the second, should be considered a preparatory stage that influences the outcome of later SFBT implementation.

##### Barriers

###### Role clash

This theme describes how participants' medical training conflicted with the SFBT and required a significant level of investment to unlearn the medical approach in order to implement it: ‘It's really difficult. You've been doing something for 40 years and you suddenly (have) to change an entire way of running a clinic’ (Participant 5).

It was also described that the approach required a change from their typical professional language they were accustomed to, which caused discomfort: ‘I might use some of the phrases, but then I'd be uncomfortable using it because it's not what I normally say or not part of my normal language’ (Participant 5).

##### Facilitators

###### Connecting with SFBT

This theme describes how participants formed a personal connection with the SFBT model. There were two main causes of such a connection: (1) SFBT as a much‐needed alternative approach in their role and (2) a personal fit to individual values.

With regards to the first cause, several participants described a sense of relief that there was an alternative model to the medical approach for supporting clients with long‐term complications:

It (SFBT) gives you a more positive approach to something that potentially has been weighing you down because you can't fix it, you can't make it better… you can't do it in Diabetes, you can't fix ‐ we can't make it better, but we can find techniques to make it better (Participant 4).

Participants further described feeling that the medical model was too problem‐focused, which they considered unhelpful to clients and often led to a sense of feeling ‘drained’, ‘demoralised’ or ‘guilty’. SFBT became a ‘solution’ to this (Participant 4) and another described a sense of desperation for anything to help support their clients as they had ‘nothing else’ (Participant 3), which facilitated motivation to try the SFBT approach.

Some participants were motivated to use SFBT through a personal connection, where they described a sense of yearning for more empathy and feelings that were lacking in the medical approach, and felt relieved that there was a recognised approach that incorporated this:

This (SFBT) is what I've been looking for… I want to be able to address them in this way. I have a platform that says that this is a recognized way of doing things. I'm not just chit‐chatting, I'm doing so much more than that. I think that was something I was very motivated by (Participant 7).

###### Witnessing successful SFBT practice

Several participants commented on the utility of observing colleagues use SFBT in practice prior to the training. They described how this provided them evidence of the effectiveness of SFBT and how seeing certain ‘success stories’ kept them going, despite causing them discomfort and investment:

Because I know that it works, I've seen it work. I still feel that it's worth doing because it can make a difference where other things haven't made a difference. I suppose it gives me the motivation, doesn't it, to go on and keep thinking of that young man… (Interview 9).

They also described the reputation of SFBT from trusted colleagues or team narratives as evidence of the credibility of SFBT, which motivated them to attend the training:

They talked really positively at how they found that (training), they were already starting to implement or make changes within the team so it was already here and it was already talked about and I was really interested in attending the training myself (Participant 8).

However, if such proofs weren't available, people reported being much more sceptical of the validity and applicability of SFBT (see Absence of Successful SFBT Practice in Figure 1).

#### Stage 2: During training

##### Barriers

###### Negative preconceptions

This theme is based on the sceptical preconceptions about SFBT that participants brought into the training. This was mainly related to doubts about its applicability in a medical context and their own ability to use it, not being psychological practitioners. For instance, one mentioned how the training challenged their preconceptions:

It was really useful in demonstrating that it doesn't have to be this huge intervention, that you have to be the world's best psychologist to use it and it can be quite adaptable depending on… the time that you might have (Participant 6).

##### Facilitators

###### Role modelling through role‐plays

Participants described the approach of the trainers in discussions and role‐plays as being an important facilitator for motivation to engage in training and in giving future confidence to implement SFBT.

For example, participants reported that the trainers often provided role‐playing space and time, and in debrief discussions highlighted the importance of silence as a tool to allow the client to lead and feel listened to. This also helped reassure participants: ‘And I picked up…from the training, all from watching the way he (clinical psychologist) works…that silence is OK and it's OK to take a minute and OK to not rush in with the next question’ (Participant 8).

###### Realistic practice opportunities

Most participants emphasised the importance of practical opportunities in training in making them feel more interested and engaged. Several participants particularly appreciated the opportunity to bring in examples from their own professional practice in role‐plays and scenarios, making them more relatable, which reinforced a sense of trust in the psychologist and the SFBT being more applicable to their client population and service context:

I don't think you can do solution focused (training) by giving someone “here's a card and you play this person now”. I think you have to relate because you gotta go into your own feelings. I think you've got to relate it to something (in) real life (Participant 4).

#### Stage 3: After training

##### Barriers

###### Problem‐oriented clients

Participants often stated that clients themselves were a barrier to their confidence of implementing SFBT.

For instance, participants described how they often felt pulled into using a medical approach whenever a client was focused on an immediate problem and requested a ‘quick fix’. Participants would in this case resort to use more of a medically expert‐led approach:

We said “What do they want?” And the response was just “not to have epilepsy” and then you go “well, what else” or try and redirect the conversation and she'll just come back to “I just don't wanna have epilepsy”. And she was just so focused on it and we couldn't get to anywhere else (Participant 3)

###### Lack of time and opportunity

This theme relates to the participants' perceptions and experiences of insufficient time and opportunities to use SFBT, affecting their motivation and confidence.

With regards to time, most participants described how SFBT requires more time than the medical model due to the need for exploration. For instance, one participant mentioned how initial conversations require more time for ‘personal information’ about the client to develop ‘good rapport’ (Participant 6). As the staff were often contracted to provide 20–30‐min clinics, such a requirement meant that explorative conversations were often disrupted, requiring more follow‐up sessions. Coupled with staff reporting such conversations being more difficult to return to as well as other competing demands, they would often miss opportunities to explore the clients' ‘main’ difficulties through the use of SFBT (Participant 7).

Moreover, SFBT was also perceived as needing more time for clients to feel comfortable in taking the lead in conversations. For instance, one participant mentioned how clients needed to go out of their own ‘comfort zone’, referring to the challenge of doing something new. This then required clinicians to ‘draw them out’ in conversations, which was time‐consuming: ‘I'm asking people to step out of their comfort zone for a lot of things and come into my world try something different’ (Participant 7).

Finally, it was mentioned that the participants needed more time at first when they were not yet confident with the use of question and other SFBT techniques. This was described as being exacerbated by a lack of opportunity: ‘At the start it was very shaky, forgetting the key questions…and forgetting where to… direct it and that's still there because we don't practice it every day, we don't even practice it every week’ (Participant 3).

###### Unrealistic expectations of SFBT

This theme relates to several assumptions and expectations that were formed about SFBT after training, based on the preconceptions developed before and during training. It was evident that participants had formed different ideas of what SFBT entails and thus also differing expectations of what they should be able to offer. For instance, several participants wanted their clients to identify comprehensive goals by the end of an initial 20–30‐min session. This is in contrast to participants who acknowledged time constraints and the remit of their non‐therapist role, working instead for their first sessions to ‘just develop rapport’ or focusing on one goal at a time (Participant 3). Participants with an unrealistic understanding of SFBT were more likely to resort to a medical approach of ‘fixing’ by providing advice or education rather than supporting the client to lead their own conversations due to time constraints. They also appeared more likely to conclude that SFBT was a context‐dependent ‘tool’ that in these cases was ‘not working’ (Participant 5).

###### Service pressures

The final barrier relates to the external pressures in health care services. For instance, several participants talked about how their services' performance goals often pulled participants into the comfort zone by shifting the team's focus and reducing individual motivation:

So it's very easy to get drawn into pushing your patients for your own or the Trust's gain…when we're published in that National Audit, “where do we sit, what number are we in the country?”. So I think it's more the target that drives you to go back to your medical model. (Participant 4)

##### Facilitators

###### Proactive pursuit of support

This theme related to how participants who were more connected with SFBT pre‐training were later more proactive and motivated to find ways to improve their SFBT learning despite external challenges. For instance, several participants described that they had proactively requested a supervisor (Participant 7), sought advice from staff members more experienced in SFBT (Participants 1, 2 and 7), or established discussion groups with their colleagues (Participants 1, 3, 4 and 9).

###### Support networks

This theme describes how supervisors, colleagues, and the teams supported staff to use SFBT.

One of the main contributions of the support network was around providing staff regular ‘reminders’ after training in order to maintain confidence and prevent depletion over time. Participants identified that regular multidisciplinary team (MDT) meetings and supervision were particularly helpful in providing them a reminder of what they were already doing, which helped them feel reassured and more confident: ‘The supervision or the MDT…make me feel a little bit more confident, that little boost to know that something's worked…or something's going OK and just makes us all feel a little bit better I suppose’ (Interview 8).

The MDT meetings were particularly appreciated as it provided a sense of collective purpose and a sense of ‘doing it together’ as a team (Participant 8) but also a sense of continuity by discussing SFBT cases regularly and develop more confidence: ‘I think even just speaking together as a team, bouncing off ideas sort of one that thing that's worked for one patient, you might sit there and think…’oh, yeah, that might work for them’ (Participant 9).

Staff also reported they benefitted the most when MDT meetings were delivered in an SFBT way by also focusing on what the staff members were doing well:

I like the way we do that “pleased to notice” because sometimes I think you can get bogged down with what's not going well and you forget to focus on the things that have been good… It will give you a “right, ok, I'm prepared to give that a go”. (Participant 4)

###### Realistic expectations of SFBT

In contrast to unrealistic expectations, realistic expectations helped participants form more realistic goals and acted as a buffer against systemic barriers and challenges by making them more confident. Findings indicated that such expectations were facilitated by supportive teams as well as good role modelling from the training.

## DISCUSSION

To our knowledge, this study was the first to construct a model which attempts to formulate the deeper mechanics of SFBT implementation, looking at how various facilitators and barriers interact to influence SFBT practice for non‐psychology health care professionals over time. Our study identified various facilitators that could counteract potential barriers to implementing practice.

Firstly, it was found that participants who had developed a personal connection with the SFBT model were more likely to proactively seek ways to increase and improve their use of it in future practice. This was predominately in the form of seeking support from their individual colleagues, teams, or supervisors. Furthermore, this was particularly apparent for staff members who did not already have regular clinical supervision. This suggests that establishing a connection and believing in the efficacy of SFBT could be an important buffer against common barriers reported in the literature, particularly around lack of supervision and managerial support (Smith, 2011; Stark et al., 2018). Although connecting with SFBT's ethos has been regularly reported among nurses (Bowles et al., 2001; Stark et al., 2018; Wand, 2010) and has been suggested as a possible factor in relation to training transfer (Cunanan & McCollum, 2006), this study provides a novel explanation of how it might contribute to SFBT implementation.

Secondly, staff who were provided with regular reminders and reassurances within their teams were better able to maintain their use of SFBT post‐training. Clinical supervision, peer support and multidisciplinary team meetings all served this function too. Interestingly, however, clinical supervision was not seen as an essential component for training transfer, which contrasts with previous research (e.g. Hosany et al., 2007; Ferraz & Wellman, 2009). Instead, this study highlighted the importance of a broader, supportive SFBT culture where staff could engage in regular solution‐focused‐informed discussions with other team members about clients.

Finally, participants also reported benefitting from reflecting on positive changes noticed in their own personal and professional lives, which encouraged them to engage in more strength‐based thinking more naturally. In these contexts, staff were often able to ask others questions about clinical cases, hear other people's experiences and receive feedback around their own progression using SFBT. This gave them the confirmation they needed to feel more confident to continue using SFBT in practice. Several other studies have indicated that a solution‐focused friendly environment is conducive to SFBT training transfer, reporting that it provides staff regular opportunities for sharing ideas through a shared language (Cunanan & McCollum, 2006; Seko et al., 2021). It might therefore be that in the presence of such supportive teams, individual clinical supervision might be less of a necessity for SFBT training transfer; and that team‐based supervision might be a more cost‐effective investment.

Our study also identified a number of barriers to implementing SFBT. One particular barrier of importance related to the clinicians' transitioning from a medical, problem‐orientated approach to a strength‐based approach. This echoes previous research reporting that staff often struggle with the required shift in their mindset, language, and approach (Cunanan & McCollum, 2006; Simm et al., 2011), often finding themselves slipping back into the approach they are more familiar with (Smith, 2011). Going further, however, this study suggested that this was due to the medical approach representing a sense of safety, where transitioning was perceived to involve many sacrifices and a sense of loss. For participants, the sacrifice was in the form of the confidence that came with the skills associated with a familiar approach. This was therefore perceived as a high price to pay and would affect their interest, motivation, and confidence to engage in the training and use of SFBT.

These results can be understood in the context of ‘Conservation of Resources’ theory (Hobfoll, 1989), which suggests that the motivation to prevent the loss of resources is greater than the potential gain of new resources. This suggests that in order for staff to be motivated throughout and following the training, they need to be ‘convinced’ that the transition towards using SFBT will be worth the loss of confidence and the cost of the required investment. Staff reported needing ‘proof’, such as seeing the therapy work in practice, in order to feel motivated to attend and engage in the SFBT training. This could be interpreted in the context of research suggesting that some staff present with initial scepticism about SFBT, perceiving it as ‘naïve’ as it does not take account of the complexity of the client's problem and past (Cunanan & McCollum, 2006). Seeing SFBT successfully work in practice was considered as important evidence to the contrary. This overlaps with findings in the SFBT training literature suggesting that observing successful implementation was experienced as an ‘aha!’ moment, which is important in facilitating future investment in the model (Stark et al., 2018). It might also be that observing and engaging in role‐plays that were more ‘realistic’ also functioned as an ‘aha’ moment.

Another reason for scepticism was the staff members' own ability to use SFBT. Perceived self‐efficacy has been found to correlate highly with and so may play an important role in skill transfer (Blume et al., 2010). The findings of our study suggest that the trainers' modelling of SFBT implementation via role‐plays was seen as increasing trainees' self‐efficacy. The importance of role‐play and live demonstrations has previously been highlighted (Cunanan & McCollum, 2006; Stark et al., 2018), but there has been a lack of research indicating how staff benefit from it. Our study illustrated how staff present with various negative preconceptions about their ability to deliver therapy, which if left unchallenged led to the formation of unrealistic expectations regarding what they were supposed to offer following the training. These unrealistic expectations could in turn lead to negative conclusions about the utility of the model. Our study highlighted that the trainers' demonstration provided trainees with a realistic idea of what an SFBT intervention would normally look like, in line with research recommending clear goal setting as part of any training in order to counter unrealistic expectations (Suleiman et al., 2017). For instance, one of the aspects of the demonstrations was the use of silence, which helped staff challenge the idea that they always needed to have the answers.

Our study also highlighted that the restrictions on staff time can negatively impact the implementation of SFBT. This has also been reported by other researchers in the field (e.g. Smith, 2011); however, the findings of our study provided further insight into how this might occur. For instance, participants in this study reported two aspects of an SFBT conversation that were seen as time‐consuming, namely (1) asking questions about clients' lives outside the medical problem and (2) supporting the client to lead the conversation. The latter was perceived as in sharp contrast to the medical ‘fixing’ approach where the clinician would often take the lead in providing advice and education. Participants reported that at the start, when confidence was low, using question techniques to guide the client was difficult and even more so with many of their clients who were also unfamiliar and uncomfortable with this way of conversing, requiring more time for clinicians to ‘draw them’ into such a conversational dynamic.

When these experienced difficulties were combined with unrealistic expectations of the outcomes of a session, this could lead participants to conclude that the model was just not usable in these situations. Somewhat ironically, however, our findings suggested that supporting staff to use silence as a tool might help them achieve SFBT goals more quickly through facilitating realistic expectations and increasing self‐efficacy. This therefore suggests that although time can be a barrier, this is highly dependent on the staff members' expectations, preconceptions and confidence.

### Study Limitations

Due to the scale of this project, recruitment for this study was halted once theoretical sufficiency was achieved. Whilst this is recognised as a valid approach (e.g. Corbin & Strauss, 2015; Dey, 1999) which enables the production of a clear, helpful model, collecting further data until saturation (e.g. Saunders et al., 2018) may have identified additional relevant factors that would have increased the explanatory power of the findings. For example, the sample in this study was relatively homogenous, which might limit the reliability and transferability of the data. More theoretical sampling may have generated data from a wider pool of staff experience. For instance, most of the staff in this study were female specialist nurses and therefore lacked representation of gender as well as other professionals who have been trained in this way. As indicated by previous research, nurses might be particularly motivated or potentially better equipped to use SFBT compared to other professions and might have found the transition to use SFBT easier. In addition to this, several of the participants also worked in services that had already embraced a shift towards more strength‐based and solution‐focused thinking, meaning they were likely more receptive to the training than staff in other health care services.

There was also a considerable degree of variation between the training programmes the staff attended. Therefore, the study could have benefitted from more demographic information on the participants and training programmes such as years of work experience, number of attendees, and nature and style of the training.

### Clinical implications and recommendations for clinical practice

Several recommendations were identified for clinical psychologists and other trainers when providing brief SFBT training in health care and medical services. First, trainers should ensure that live demonstrations and role‐plays are specific to the relevant service and client group (Seko et al., 2021), taking account of the challenges of using SFBT in time‐restricted clinics and risk‐related conversations. It might be helpful for staff members to bring in their own cases for discussion and role‐plays, as well as facilitate group discussions around common challenges. Furthermore, clinical psychologists should consider including ‘imperfections’ in their demonstrations, such as silence, to increase trainee self‐efficacy and challenge unrealistic expectations.

Second, trainers should consider the importance of staff observing successful interventions using SFBT. Optimally, staff would be able to observe a trained SFBT practitioner delivering SFBT before they attended training, in order to facilitate motivation to attend and engagement in the training. However, as this might not always be feasible, an alternative might be to include video of successful interventions in the training in order to provide staff with a sense of evidence that SFBT is worth their investment.

Finally, trainers who are working within or into the service trainees are drawn from should invest time and effort in building a solution‐focused friendly environment (Stark et al., 2018). This could be facilitated by ensuring that newcomers are provided with SFBT training and shadowing opportunities of SFBT in practice. They should also consider facilitating regular MDT meetings as group supervision of SFBT, providing staff opportunities to discuss and reflect upon cases where using SFBT principles have been attempted. In this study, staff also benefitted from MDT discussions that invited them to notice what was going well, which is echoed by SFBTA practice guidelines for practitioners to identify ‘what they are already doing that is working’ (SFBTA, 2012). It is likely that such strength‐focused meetings further facilitate strength‐based thinking and subsequent implementation.

### Research implications

This was, to the authors' knowledge, the first study using a grounded theory approach to explore the experiences of medical staff receiving brief SFBT training. Although some of the results from this study echoed those of previous studies (Cunanan & McCollum, 2006; Seko et al., 2021; Smith, 2011; Stark et al., 2018), several novel findings were identified that could benefit from further exploration.

First, several factors were identified that facilitated a sense of interest and motivation to attend the SFBT training. Whilst in our study we did not identify specific differences between staff members who had volunteered for the training versus those who were required to attend, in order to develop deeper insight into the factors that might be involved in motivation to attend, further qualitative research interviewing staff who decided not to attend the training would be beneficial. Being able to more accurately identify why staff choose not to attend SFBT training might be particularly important for clinical psychologists attempting to build more solution‐focused friendly teams and service cultures.

Second, this study indicated that one‐to‐one clinical supervision sessions might not be essential in maintaining training transfer for all staff within a context of a solution‐focused environment offering regular solution‐focused MDT meetings. Future research would benefit from exploring this concept further by comparing staff experiences with and without supervision in health care environments.

Third, this study replicated previous research in suggesting that individual connection with the SFBT ethos was important to facilitate further implementation. Although this study was the first to attempt explaining how the connection led to training transfer, the data of this study could not explain how it was developed. Future qualitative research should look into how this connection could be beneficial in order to support trainers to tailor courses to individual or group‐based preferences and manage various team dynamics better. This could be explored by comparing the experiences of staff with different personality profiles (e.g. using the Big Five Inventory (BFI ‐John et al., 1991)) and professional roles.

## CONCLUSION

Our study suggests that health care staff need to be convinced of SFBT's utility and their ability to implement it before they are able to transition out of using the medical model. If trainers can ensure that those attending courses have observed demonstrations of successful implementation of the model before training, this may facilitate motivation to attend and engage in subsequent training. Modelling realistic SFBT implementation that is relevant to the specific service will likely help negate unrealistic expectations. Finally, trainers who can invest time to develop solution‐focused friendly environments can counter the harmful impacts caused by a range of systemic barriers and facilitate long‐term confidence and training transfer.

## AUTHOR CONTRIBUTIONS

**Haakon Juul:** Investigation; methodology; formal analysis; writing – review and editing; writing – original draft; visualization. **Dominic Bray:** Supervision; writing – review and editing; conceptualization. **Ian C. Smith:** Conceptualization; supervision; formal analysis; visualization; writing – review and editing; methodology.

## CONFLICT OF INTEREST STATEMENT

The authors have no conflicts of interest to report.

## Supporting information


Data S1.

## Data Availability

The authors cannot make the data from this study publicly available as to do so would compromise the anonymity of participants and contravene the conditions under which ethical approval for conducting the study was granted. This is in turn due to the personal and in‐depth qualitative nature of the data collected.
