# Supplementary material for: What works? A grounded theory investigation of training non‐psychology staff in using Solution‐Focused Brief Therapy
Source: Psychol Psychother. 2025 Aug 17;99(1):60–79. doi: 10.1111/papt.70009 (PMC12905523; doi:10.1111/papt.70009)
Supplement: Supplementary file 1 — Data S1. [file PAPT-99-60-s001.docx]

**Supplementary Materials**

1. **Example of memo writing and field notes**

| Interview 2 |
| --- |
| Felt more confident in this review than last one, but did also feel there were some tentativeness from them around the negatives around the training and I’m wondering how much of that could be because the trainer hey had is my field supervisor. I think it was really helpful to re-iterate confidentiality and limits of it and how they could be completely honest. I think in future interviews I will re-iterate this in the introduction and how their experience (whether good or bad) would be just as relevant as it would tell us what works and doesn’t (i.e. it would help regardless).  This interview had some interesting reflections around the power of the service and how the any in the service focus could cause a shift in the mindset? Talked about how they felt their contractual obligations and prioritisations meant that SFT and ‘exploration’ was perceived as difficult to implement at times. Could maybe explore more in future interviews.  Mentioned SFT as a tool as did interview 1. Non-psychology staff using SFT as a tool and need assessment skills of whether to use SFT or not as it is not applicable in all situations (and often falls outside the trained scenarios) – beneficial for training around when to use it and when not to? Might be again a difference between therapists and non-psychology staff– they cannot use it as a whole framework, partially because they would need many other techniques/skills but also because it might not suit the situation? Also, a clinical psychologist might not fully understand the non-specialist role and obligations and thus might be a communication barrier between the facilitator and trainee.  Discussing and working closely with the service managers and also the trainees. Also talking about importance of scenarios like with interview 1, would need to explore further to see if it is appreciated – avoid biased questions around this in the future – discuss in supervision, particularly difference between being biased vs. reaching theoretical saturation and using theoretical sampling to explore more of what is emergent from data. Might be possible that this might relate to future recommendations for training, how training needs to be more relevant and ‘relatable’ and also to communicate certain barriers for using therapy in general – dedicating a session of ‘what are some barriers to prevent you from using SFT?  Non-psychology staff experience very different situations than therapists doing the sessions: nurses might respond to an urgent crisis – the expected role is not to offer therapy in this context. They then need to assess whether it would be helpful or well received. For therapists, clients often come to them and expect therapeutic input -> expectation important – theme from 2 as well -> another difference is therefore that patients coming to see a nurse do expect invasive physiological procedures but not invasive psychological procedures (e.g. questions) -> this is a huge difference.  Something around the problem having to be acknowledged first before SFT work can be done – if fixated on the problem – how can one get to the strengths? Looking for strengths and resources require a broader perspective and search – a story that contains other elements than one is used to. A child who has a medical diagnosis as well as a continuous physiological trigger would likely fixate -> potential barrier for people with little experience. Something about patients identifying their own goals – not advice, ‘doing it  themselves’ -> why is this important? Also, it was mentioned that this does not always work – why? Advice, recommendations and providing solutions/answers more useful – is this outside of the SFT framework? How do people perceive something as advice and solutions vs giving people a chance to identify it themselves – is there anything that stops staff from helping clients realising it -> it was mentioned that time and some form of ‘fixation’ (their word) on a problem or not knowing their own goals. What about this is challenging?  Remembering that the interviews are about identifying what the trainees got from the training – this includes how they understood SFT. It could be discussed then how this understanding led to other consequences, such as how it was used or motivation etc. Their understanding of SFT and how to use it is possibly quite important.  Also seems like first impressions and reputations had an impact on interest and motivation to attend and engage in the training -> but does it have any further impact beyond this? Does it act as a buffer against poor or mediocre training or enhancing the positive experience? How much and what other factors might be involved?  Upon reflection, I think I could have asked more questions around the causes of the changes they experienced – too much focus on what the changes were and could be helpful to consider more around this in future interviews. |

1. **Example of how model was developed from quotes, codes, memos and theoretical categories**

| Participant | Data (Quotes) | Initial Codes | Focused Codes | Theoretical Codes | Memos/Notes |
| --- | --- | --- | --- | --- | --- |
| Interview 3 | P: So the diabetes team was using it and they were quite encouraging, which *redacted* (trainer) works in. And then our *redacted* (Consultant Doctor) they are a big advocate for it. So they got into before *redacted* (trainer) even came here. They run a clinic once a week with children who come to A&E with mental health problems regularly and stuff like that. And they use solution focus in their conversations to talk with these families and stuff. So we've already got that kind of stuff going on here, yeah, which gives you a good insight. But then also we've got - obviously, *redacted* (trainer) working here was also as a big advocate for it. So we're quite lucky in what we already had in terms of understanding how it's going to be beneficial, I suppose. | Separate team being encouraging for them to use it  Consultant doctor also advocating for it who also used it regularly.  Feeling that other teams and colleagues using it gives the team insight about the model  Trainer as an advocate as well  Feeling this helped them understand how SFT would be beneficial prior to the training | Teams giving them insight into benefits of SFT before training | Positive reputation acts like evidence for people to feel more interested, which motivates them and facilitates an increased willingness to invest | Wonder if this in a way is the start of the formation of a relationship with the model or the ethos of the model where basically this represents the first impression. I am curious then about how impactful this actually is – is this shared with other staff. Will be interesting to keep in mind in future interviews but important to keep mind open as it might not be important or an influential factor for some people at all (or somewhere in between). |
